# Mental health service use and costs associated with complex emotional needs and a diagnosis of personality disorder: analysis of routine data

**DOI:** 10.1192/bjb.2023.41

**Published:** 2024-04

**Authors:** Joseph Botham, Alan Simpson, Paul McCrone

**Affiliations:** 1 Institute of Psychiatry, Psychology and Neuroscience, King's College London, UK; 2 Institute for Lifecourse Development, University of Greenwich, UK

**Keywords:** Complex emotional needs, personality disorders, service use, cost, economics

## Abstract

**Aims and method:**

We aimed to estimate the costs of care for people with a personality disorder diagnosis and compare service use and costs for those receiving specialist input and those receiving generic care. Service use data were obtained from records and costs calculated. Comparisons were made between those who received care from specialist personality disorder teams and those who did not. Demographic and clinical predictors of costs were identified with regression modelling.

**Results:**

Mean total costs before diagnosis were £10 156 for the specialist group and £11 531 for the non-specialist group. Post-diagnosis costs were £24 017 and £22 266 respectively. Costs were associated with specialist care, comorbid conditions and living outside of London.

**Clinical implications:**

Receiving increased support from a specialist service may reduce the need for in-patient care. This may be clinically appropriate and results in a distribution of costs.

It has been estimated that about 8% of the population meet the criteria for a diagnosis of personality disorder.^1^ The concept of personality disorder is contentious and the term ‘complex emotional needs’ may be preferred, although it is recognised that much published literature still uses the term ‘personality disorder’.^2^ A diagnosis of personality disorder has been shown to be associated with high rates of comorbidity and resource use.^3–5^ Estimates of the cost of care for people with a diagnosis of personality disorder vary substantially. In England, these were estimated at £7.9 billion in 2006, with projected costs of £12.3 billion by 2026.^6^ Most studies that have estimated care costs have done so using data from trials, surveys or hospital data.^4,5,7^

Feedback from a stakeholder and lived-experience group workshop suggested a diagnosis of personality disorder often resulted in people being denied the services/care they needed, which would result in poorer outcomes with an impact on long-term cost.^8^ Understanding the factors that drive costs is useful for policy and decisions makers to make informed choices on how best to allocate healthcare resources.

The aims of this paper are to (a) compare the service use and costs for people with a personality disorder diagnosis between those who use specialist personality disorder services and those who do not, (b) make comparisons for the two groups before and after they received a diagnosis of personality disorder and (c) identify individual-level predictors of care costs following diagnosis.

## Method

### Setting and sample

Data were obtained from the Clinical Records Interactive Search (CRIS) database of the South London and Maudsley NHS Foundation Trust (SLaM). SLaM covers a geographical area of four South London boroughs with a combined population of around 1.3 million people; it also provides specialist services for those from other areas and nationally. The geographical area is relatively deprived, although with substantial variation within boroughs. The CRIS database was launched in 2008 and includes data going back to 2006 from electronic patient records across SLaM, and anonymised information are made available for research studies.^9^ The data include patient background demographic and clinical information, and records of in-patient stays and contacts with clinical staff. The database also enables machine learning methods to be used to search through free-text information. Ethical approval is in place for records to be used for research, and approval for individual projects is given by an oversight committee.

We used an approach for identifying the relevant sample that had been used in previous work by Fok et al.^10^ The CRIS database was searched by Fok et al through a combination of text field and natural language processing methods for a new sample of individuals who had been given a diagnosis of a personality disorder (excluding antisocial personality disorder) between 1 April 2008 and 31 March 2016.^10^ The search terms used were related to ‘personality disorder’ and types of personality disorder, as well as ICD-10 codes (F60.0–F61; but excluding F60.2).^11^ The text field searches were run on primary and secondary diagnoses data, and natural language processing was used to search patient notes and other unstructured texts stored on the database (such as risk assessments and care plans). Individuals were excluded from the analysis if they had no face-to-face contact with SLaM services during the period of interest – it is worth pointing out that the data is pre-COVID19 and ‘no face-to-face contact’ would mean an individual had not had any contact.

### Demographic variables

Variables relating to background characteristics were extracted from the database. These included date of birth, date of death (if applicable), gender, ethnicity, relationship status, housing status, truncated postcode and Index of Multiple Deprivation (IMD) score. IMD scores rank the relative deprivation of small neighbourhoods and areas and are used as an indication of socioeconomic deprivation.^12^ IMD scores range from 1 to 32 844 (which reflects the total number of areas), and a lower score indicates a higher level of deprivation.

### Clinical variables

Clinical data were extracted on primary and secondary diagnoses and Health of the Nation Outcomes Scales (HoNOS) data.^13^ Primary and secondary diagnosis data were extracted and transformed into binary variables for individual and groups of conditions. A truncated search was used to identify strings of text that matched either the code, name or alternative descriptions outlined by ICD-10 descriptions, and the full code is available from the authors upon request. Individual variables were used for each code for specific personality disorders (F60.0–F60.9 and F61). Binary variables were also generated for severe mental illness (schizophrenia, bipolar disorder and other psychoses), other mental illness, alcohol/drug use and intellectual disability. Variables were also generated for use of the terms ‘severe’ and ‘self-harm’. HoNOS scores, recorded closest to the date of diagnosis (one month either side), were also extracted.

### Service use and costs

Service use data were extracted on the number of face-to-face community team contacts, bed days on non-forensic wards and bed days on forensic wards, for each individual between 1 April 2008 and 31 March 2016. Preliminary data extractions showed that the cohort had been in contact with over 900 community teams, so teams were allocated into groups of similar function. Each group of teams, non-forensic bed days and forensic bed days were assigned an appropriate unit cost from the 2016–2017 NHS reference cost schedule.^14^ This is an annual compendium of average cost information derived from all NHS Trusts in England. Specific costs relate to specialist in-patient and out-patient mental healthcare. The ‘best-fit’ cost was used, given that the local service definitions were far more specific than those in the reference cost schedule.

### Analysis

Individuals were grouped based on the date of first contact with SLaM and the date of the initial diagnosis of personality disorder. The groups included in the analysis were those who (a) had been in contact with SLaM and had a diagnosis predating 1 April 2008; (b) had been in contact with SLaM before 1 April 2008, but had not received a diagnosis until after this date; (c) had a first contact with SLaM after 1 April 2008, but did not have a recorded diagnosis of a personality disorder until later on in the study window and (d) had a first contact with SLaM after 1 April 2008 and had a diagnosis of a personality disorder at this time.

Comparisons of service use and costs were made between individuals who at some point during the study period had contact with specialist services for people diagnosed with personality disorder, defined as contact with complex emotional needs teams or receipt of dialectical behavioural therapy (DBT), and those who received non-specialist care. We also made comparisons between the period before a diagnosis being given and the period following this. These comparisons were descriptive, and we only tested total costs for statistical significance.

Following the descriptive comparisons, we used a regression model to identify predictors of service costs during the post-diagnosis period. The independent variables in the regression model included demographic characteristics, diagnosis, HoNOS score and comorbidities. Variables were entered either in binary (with 1 indicating the condition was met and 0 indicating otherwise) or continuous form. The set of diagnosis dummy variables were not mutually exclusive or exhaustive, and therefore a reference category was not required. A simple ordinary least squares linear model was used. Although cost data are usually skewed, large sample sizes such as this mean that results are robust to violation of the assumption of normally distributed residuals.

Analysis was carried out with Stata/MP version 15 for Windows 16.

### Ethical approval and consent

The CRIS database has been approved for secondary analysis by Oxford Research Ethics Committee C (approval number 18/SC/0372). Data are anonymised and consent was not applicable. Individuals were able to have their records excluded from the CRIS system.

## Results

Data on 9345 individuals were extracted from the CRIS database. Of these, 511 did not have any contact with services during the study period. A further 687 were excluded as they were identified as having an F60.2 diagnosis (antisocial personality disorder). The final sample size was 8147 individuals.

Table 1 shows the sample characteristics. About two-thirds of the sample were female and a similar proportion were White British. Nearly three-quarters of the sample were single. Most had a Greater London postcode (87.73%). Housing status was poorly reported. When comparing specialist care and non-specialist care groups, there were differences in gender (specialist group: 69% female; non-specialist group: 59% female) and location (specialist group: 96% from London; non-specialist group: 84% from London). There were significant, albeit small, differences in age, IMD score and mean number of F60 diagnoses. The specialist care group had a higher proportion of F60.3 diagnoses (‘emotionally unstable’) (72%) than the non-specialist group (56%).

**Table 1:** Cohort characteristics

	Specialist treatment (*n* = 2798; 34.4%)	Non-specialist treatment (*n* = 5349; 65.7%)	Total (*N* = 8147)
Age, years, mean (s.d.)	32.9 (12.3)	34.6 (15.1)	34.0 (14.2)
Female, %	69.0	59.1	62.5
British, %	62.9	64.0	63.7
Relationship status, %			
Single	73.3	70.1	71.2
In a relationship	11.8	9.9	10.6
Divorced/separated	10.0	8.8	9.2
Widowed	0.6	1.7	1.3
Unknown	4.3	9.6	7.8
Housing status, %			
Homeowner	1.8	1.2	1.4
Tenant	12.5	10.3	11.1
Residential care	0.6	1.1	0.9
Homeless	1.0	1.8	1.6
Unknown	84.1	85.7	85.1
London, %	95.7	83.6	87.7
Index of Multiple Deprivation score, mean (s.d.)	10 075 (6307)	10 454 (6565)	10 321 (6478)
F60X diagnosis, mean (s.d.)	1.3 (0.6)	1.1 (0.5)	1.2 (0.5)
F60.0 ‘Paranoid’	71 (2.5)	192 (3.6)	
F60.1 ‘Schizoid’	22 (0.8)	89 (1.7)	
F60.3 ‘Emotionally unstable’	2025 (72.4)	3011 (56.3)	
F60.4 ‘Histrionic’	22 (0.8)	28 (0.5)	
F60.5 ‘Anankastic’	87 (3.1)	92 (1.7)	
F60.6 ‘Anxious (avoidant)’	48 (1.7)	78 (1.5)	
F60.7 ‘Dependant’	121 (4.3)	173 (3.2)	
F60.8 ‘Other (specified)’	54 (1.9)	100 (1.9)	
F60.9 ‘Other (unspecified)’	984 (35.2)	1934 (36.2)	
F61 ‘Mixed’	247 (8.8)	401 (7.50	

### Service use

Table 2 shows the numbers and percentages of the sample that were using specific services before and after a diagnosis was recorded. The data are reported by groups defined by whether they had used specialist services for people diagnosed with personality disorder or complex emotional needs at some time during the study period. For those who did use services at least once, the mean and s.d. number of contacts is reported.

**Table 2:** Use of services pre- and post-diagnosis by groups defined by receipt of specialist care

Service	Pre-diagnosis				Post-diagnosis				Total period			
	Specialist		Non-specialist		Specialist		Non-specialist		Specialist		Non-specialist	
	Service use, *n* (%)	Number of contacts, mean (s.d.)	Service use, *n* (%)	Number of contacts, mean (s.d.)	Service use, *n* (%)	Number of contacts, mean (s.d.)	Service use, *n* (%)	Number of contacts, mean (s.d.)	Service use, *n* (%)	Number of contacts, mean (s.d.)	Service use, *n* (%)	Number of contacts, mean (s.d.)
In-patient services												
Bed days (non-forensic)	699 (24.1)	57.6 (143.4)	1141 (22.3)	87.1 (203.4)	957 (34.2)	89.9 (181.6)	1484 (27.7)	120.4 (302.9)	1188 (43.5)	106.3 (211.6)	1811 (33.9)	153.5 (136.8)
Forensic bed days	4 (0.1)	89.3 (59.3)	66 (1.2)	310.2 (51.9)	16 (0.6)	398.7 (334.7)	120 (2.2)	691.8 (649.6)	17 (0.6)	396.2 (335.7)	130 (2.4)	796.1 (675.4)
Community services												
Adult	1737 (62.1)	23.3 (39.2)	2873 (53.7)	17.5 (41.8)	2121 (75.8)	39.6 (61.3)	3863 (72.2)	25.2 (54.7)	2464 (88.1)	50.5 (70.7)	4464 (83.5)	33.1 (66.2)
Older adult	14 (0.5)	12.9 (17.8)	165 (3.1)	20.9 (27.4)	26 (0.9)	6.8 (12.0)	228 (4.3)	22.8 (33.0)	34 (1.2)	10.5 (20.2)	260 (4.9)	33.3 (43.0)
Child and adolescent mental health services	331 (11.8)	31.7 (42.2)	536 (10.0)	16.7 (25.6)	221 (7.9)	25.3 (42.5)	469 (8.8)	21.1 (35.0)	410 (14.7)	39.2 (55.8)	744 (13.9)	25.3 (39.8)
Dialectical behaviour therapy	71 (2.5)	10.3 (12.5)	−	−	299 (10.7)	30.9 (34.0)	−	−	315 (11.3)	31.7 (34.7)	−	−
Complex emotional needs	1084 (38.7)	11.6 (40.7)	−	−	2288 (81.8)	35.4 (82.6)	−	−	2612 (93.4)	35.8 (83.6)	−	−
Forensic out-patient	52 (1.9)	3.3 (6.6)	176 (3.3)	19.2 (34.1)	147 (5.3)	9.6 (26.5)	414 (7.7)	39.0 (59.0)	181 (6.5)	8.7 (24.6)	476 (8.9)	41.0 (62.0)
Homelessness	9 (0.3)	8.7 (8.7)	66 (1.2)	9.9 (19.2)	12 (0.4)	11.9 (8.9)	73 (1.4)	20.2 (32.7)	16 (0.6)	13.8 (13.7)	107 (2.0)	19.9 (33.2)
Addictions	240 (8.4)	19.1 (35.3)	441 (8.2)	25.8 (40.0)	388 (13.9)	20.4 (33.1)	617 (11.5)	27.6 (43.9)	520 (18.6)	24.0 (42.6)	788 (14.7)	36.1 (57.9)
Rehabilitation	69 (2.5)	13.0 (26.5)	100 (1.9)	22.9 (44.7)	132 (4.7)	30.6 (70.0)	181 (3.4)	46.6 (112.7)	177 (6.3)	27.9 (64.4)	230 (4.3)	46.6 (106.0)

In the period before a diagnosis was given, the rates of non-forensic in-patient use were similar between groups, with slightly less than a quarter being admitted at some point. However, for those who were admitted, the number of days in hospital (which may be for multiple stays) was substantially higher for the non-specialist care group. Forensic admissions were rare, although more likely for the non-specialist group. The specialist care group were more likely to have had contacts with general adult services pre-diagnosis. Interestingly, during this pre-diagnosis period, there were still around a third of the specialist group who had contact with complex emotional needs teams and a smaller proportion who had received DBT. The non-specialist care group were more likely to have had contact with older adult services, forensic community services and homelessness services, although numbers were low.

During the post-diagnosis period, the specialist care group were more likely to have received non-forensic in-patient care than the non-specialist group, but again the latter had far more days in hospital if they received this service. The numbers admitted to forensic wards were again very low, but the proportion was higher in the non-specialist group. The proportion having contact with general adult community services was similar for both groups, and this proportion had increased noticeably from the pre-diagnosis period. Not surprisingly, substantially more people from the specialist care group now had contacts with complex emotional needs teams or had received DBT. The non-specialist group were again more likely to have had contact with older adult services.

The proportions of individuals with at least one bed day were similar to those with at least one admission for both groups; there were minor discrepancies caused by dates of admission occurring before the start date, with the stay extending into the window or patients being admitted and discharged on the same day.

### Service costs

Mean (s.d.) costs per patient with at least one in-patient or community service contact for each group during both time periods are shown in Table 3. Costs of both non-forensic and forensic in-patient care were higher for the non-specialist group compared with the specialist group during each time period. Mean (s.d.) costs of in-patient and community care for the whole sample (including those with zero contacts) can be seen in Table 3. When all individuals were considered, the total costs of in-patient care were slightly higher for the non-specialist group. The difference was 36% during the pre-diagnosis period and 20% in the post-diagnosis period. The total cost of community events was substantially higher for the specialist care group, both pre-diagnosis (by 65%) and post-diagnosis (132%). These divergent findings offset each other, and so the total mean costs between the groups were very similar in each time period.

**Table 3:** Cost of services pre- and post-diagnosis by groups defined by receipt of specialist care

	Pre-diagnosis		Post-diagnosis		Whole period	
	Specialist	Non-specialist	Specialist	Non-specialist	Specialist	Non-specialist
In-patient services						
Non-forensic in-patient	28 396 (70 706)**	42 945 (100 289)	44 317 (89 539)**	59 335 (149 347)	52 408 (104 312)**	75 678 (178 304)
Forensic in-patient	12 227 (8122)	42 501 (57 795)	54 620 (45 851)	94 782 (88 999)	54 284 (45 988)*	109 069 (92 536)
Community services						
Adult	1826 (3660)*	1587 (4599)	2817 (4975)**	2002 (4929)	3712 (6154)**	2754 (6467)
Child and adolescent mental health services	7016 (10 280)**	3670 (5840)	5630 (9567)	4575 (7867)	8698 (13 066)**	5528 (8934)
Older adult	391 (520)	1142 (1778)	385 (976)*	1497 (2604)	455 (1052)**	2038 (3031)
Dialectical behaviour therapy	1006 (1226)	−	3029 (3331)	−	3102 (3397)	−
Complex emotional needs	1858 (6517)	−	5656 (13 219)	−	5726 (13 376)	−
Forensic out-patient	811 (1630)**	4764 (8466)	2382 (6560)**	9676 (14 633)	2168 (6099)**	10 177 (15 384)
Homeless	1387 (1395)	1588 (3065)	1907 (1424)	3231 (5226)	2210 (2195)	3184 (5310)
Addictions	2252 (4161)*	3049 (4722)	2402 (3907)**	3261 (5183)	2831 (5021)**	4260 (6837)
Rehab	4929 (10 068)	8713 (16 968)	11 622 (26 604)	17 692 (42 831)	10 588 (24 479)*	17 711 (40 265)
Total in-patient cost	7111 (37 503)*	9685 (50 954)	15 470 (57 448)	18 588 (85 334)	22 582 (73 694)*	28 273 (112 541)
Total community cost	3045 (7154)**	1846 (5760)	8547 (15 640)**	3678 (11 538)	11 592 (17 554)**	5524 (13 519)
Total cost	10 156 (39 251)	11 531 (53 111)	24 017 (63 990)	22 266 (88 204)	34 174 (80 316)	33 797 (116 221)

All costs are mean (s.d.) GBP, 2016–2017.

*P < 0.05, **P < 0.01.

### Predictors of cost

The multivariable regression model used to identify factors that had a significant effect on costs after diagnosis is shown in Table 4. The length of the period over which costs were measured was unsurprisingly positively associated with costs. Also as expected, the costs during the pre-diagnosis period were associated with subsequent costs. After controlling for other variables, each specialist event was found to significantly increase total costs by £82. Presence of comorbid serious mental illness was associated with average costs that were £21 553 higher than those without this comorbidity. Costs were £12 288 higher if addictions were present and £50 761 if the individual had an intellectual disability. Individuals who were usually resident outside of London had significantly higher costs than those based in the capital. No other variables were significantly associated with costs. Although various characteristics were significantly associated with costs, the *R*^2^-value was 0.13, indicating that 87% of cost variation remained unexplained.

**Table 4:** Predictors of costs (2016–2017 GBPs) during post-diagnosis period

Independent variable	Coefficient	95% CI
Post-diagnosis days	26	24 to 29
Pre-diagnosis costs	0.54	0.51 to 0.58
Pre-diagnosis days	−18	−21 to −14
Total specialist events	82	50 to 114
Age at start of study period	−78	−202 to 45
Male	−847	−4344 to 2658
Not British	−139	−3459 to 3183
Not single	−1476	−5130 to 2175
Not from London	10 628	4724 to 16 532
Index of Multiple Deprivation score	0.19	−0.06 to 0.45
F60.0 ‘Paranoid’	3118	−6188 to 12 475
F60.1 ‘Schizoid’	4496	−9574 to 18 601
F60.3 ‘Emotionally unstable’	2969	−1287 to 7277
F60.4 ‘Histrionic’	−4962	−25 125 to 15 164
F60.5 ‘Anankastic’	−3075	−13 767 to 7599
F60.6 ‘Anxious (avoidant)’	4384	−8323 to 17 075
F60.7 ‘Dependant’	465	−8039 to 8969
F60.8 ‘Other (specified)’	−2309	−14 059 to 9440
F60.9 ‘Other (unspecified)’	5653	1669 to 9637
F61 ‘Mixed’	5715	−397 to 11 827
Serious mental illness	21 553	17 224 to 25 882
Other mental illness	507	−4050 to 5065
Addiction	12 288	8075 to 16 502
Intellectual disability	50 761	24 996 to 76 526
'Self-harm' appearing as string text	3431	−7293 to 14 154
'Severe' appearing as string text	−7819	−13 147 to −2490
Regression constant term	−7037	−15 245 to 1110

## Discussion

This study used data from a large provider of specialist mental health services in South London to explore the service use and costs for people who received a diagnosis of personality disorder. The sample size was large, covered a long period, and a wide range of clinical and demographic variables were available for the analysis of predictors of cost. We are unaware of other studies that have used routinely collected data to explore these issues.

Around a third of the cohort had at some time received support from teams specialising in care for people diagnosed with a personality disorder. Those receiving specialist care were more likely to be younger, female, living in London, have a higher IMD score and multiple F60 diagnoses. They were also much more likely to have an F60.3 diagnosis (‘emotionally unstable’/’borderline’). The higher rates of women and F60.3 diagnosis are somewhat expected, as the National Institute for Health and Care Excellence guidance for borderline personality disorder recommends DBT for women who self-harm.^15^

Before diagnosis, around a quarter of each group had spent some time as an in-patient. Following diagnosis, this increased to about a third in the specialist care group. This may be seen as somewhat counterintuitive, as one might expect a diagnosis to be a step toward recovery (and typically reduced admissions). However, the number of in-patient days in the non-specialist group for those who were admitted was much greater. Although forensic care was seldom received, it was somewhat more likely in the non-specialist group. Overall, costs were substantially higher in the post-diagnosis period, but this was expected given the length of this was greater than the length of the pre-diagnosis period. Costs were similar between the two groups in each time period, but the distribution was very different. Pre-diagnosis, 30% of the specialist care group costs were accounted for by community services, and this increased to 36% following diagnosis. For the non-specialist care group, the figures were 16% and 17%, respectively.

Although these results are modest, it is not unexpected that those receiving specialist support would have a disproportionate amount of cost accounted for by community services. This may be seen as encouraging if a move toward more community-orientated care is preferred. We also found that comorbid conditions had a cost-raising effect. Again, this is what would be expected if costs reflect needs.

The study results imply that costs for those with complex emotional needs are high, and that in-patient care is a key contributor to cost. Costs are probably not reduced with specialist teams, but they can be redistributed. When assessing costs, it is important to consider other conditions that may be present.

### Strengths and limitations

The main strengths of this analysis are the large cohort size and the comprehensive view of the SLaM secondary mental health service.

There are important limitations to consider too. The use of routine data means there has been a trade-off between the quantity and quality of the data, as there is a high rate of missing or incomplete data relating to patient characteristics, which could not be imputed as the nature of its missingness is unknown. This does not appear to have affected any one group more than another, so differences between groups are still noteworthy; however, for some variables (ICD-10 codes), the occurrence within the cohort is likely underestimated.

The study is limited further by the method used to assess service use and estimate costs. A combination of ‘back-end’ data, online searches and expert opinion were used to identify the service each team provided if it was not reflected in the name. Costs in some aeras may be higher or lower than the national averages used here. However, it is unclear that this would have a major impact on the comparisons we have focused on here.

The data are drawn from a large provider of specialist mental healthcare in south London. Although this may have similarities with other services providing care in deprived inner-city areas, it is not representative of other areas. How the relationships between clinical and demographic characteristics and costs apply should be investigated in other areas.

In summary, this study has demonstrated that people with complex emotional needs who receive a diagnosis of personality disorder have high average costs, but these vary substantially between individuals. Few demographic and clinical characteristics were significantly associated with cost. There was some evidence that use of specialist services increased overall costs, but this may be entirely reasonable so as to provide support of a high quality. More information is needed on the effectiveness of specialist care to inform decision-making.

## Data Availability

Data are held by the Biomedical Research Centre at the South London and Maudsley NHS Foundation Trust, to whom applications can be made.

## About the authors

**Joseph Botham** is a research assistant at the Health Service and Population Research Department of the Institute of Psychiatry, Psychology and Neuroscience, King's College London, UK. **Alan Simpson** is a Professor of Mental Health Nursing at the Health Service and Population Research Department of the Institute of Psychiatry, Psychology and Neuroscience, King's College London, UK. **Paul McCrone** is a Professor of Healthcare Economics at the Institute for Lifecourse Development, University of Greenwich, UK.
